# Technology for Healthy Aging: Use of Electronic Communication among Older Adults

**DOI:** 10.1093/geroni/igab046.2404

**Published:** 2021-12-17

**Authors:** Rumei Yang, Yan Du, Haocen Wang, Zuoting Nie, Chumin Ji, Yun Jiang

**Affiliations:** 1 Nanjing Medical University, Nanjing, Jiangsu, China (People's Republic); 2 School of Nursing, University of Texas Health Science Center at San Antonio, San Antonio, Texas, United States; 3 Texas A&M University, College Station, Texas, United States; 4 School of Nursing, Nanjing, Jiangsu, China (People's Republic); 5 School of Nursing, Nanjing Medical University, Nanjing, Jiangsu, China (People's Republic); 6 University of Michigan, Ann Arbor, Michigan, United States

## Abstract

In the digital era, many electronic platforms have been established to facilitate patient-provider communication, such as e-mail, text messaging, and patient portal. The use of these electronic platforms is termed as electronic-communication (e-communication). E-communication has a variety of personalized healthcare functions, such as exchanging information, reviewing lab results, and facilitating patient engagement. However, little is known about the actual use of e-communication among older adults who are potentially major users of e-communication considering their high-level health care needs. Understanding their use of e-communication is critical in improving the application of e-communication in older adults. Using data from American Health Information National Trends Survey (HINTS2019-Cycle3; n=1,961; meanage =74.10, range=65-98), we explored: 1) the prevalence of e-communication use among older adults, and 2) factors affecting their use of e-communication. Variables were measured by self-reports. Weighted logistic regression with replicate weights provided by the HINTS was performed for data analysis. We found that 50% older adults reported the use of e-communication in the last year. Factors associated with higher likelihood of older adults’ e-communication use included younger age (OR=09.96, 95%CI=0.93-0.98, p<0.001), higher education (OR=4.82, 95%CI=2.32-10.02, p<0.001 for college graduate or higher), higher income (OR=1.58, 95%CI=1.05-2.38, p=0.030), comorbid conditions (OR=1.64, 95%CI=1.02-2.64, p<0.001), and having a regular provider (OR=2.06, 95%CI=1.31-3.22, p=0.002). This study provided nationally representative results demonstrating a great potential use of e-communication in older adults. Special attention is needed to focus on socially vulnerable older adults (e.g., those with older age, lower education and income, and having comorbidity).

